# Bioactivity evaluations of leaf extract fractions from young barley grass and correlation with their phytochemical profiles

**DOI:** 10.1186/s12906-020-2862-4

**Published:** 2020-02-28

**Authors:** Mamata Panthi, Romit Kumar Subba, Bechan Raut, Dharma Prasad Khanal, Niranjan Koirala

**Affiliations:** 1 Department of Pharmacy, Manmohan Institute of Health Sciences, Tribhuvan University, Kathmandu, Nepal; 2 Department of Natural Products Research, Dr. Koirala Research Institute for Biotechnology and Biodiversity, Kathmandu, Nepal

**Keywords:** Barley grass, Total phenolic content, Total flavonoid content, Anti-oxidant activity, RBC membrane stabilization activity, Brine shrimp, GC-MS

## Abstract

**Background:**

The pressed juice of Barley Grass (BG) has become very popular among people for various assumed benefits along with many testimonies of people who have been healed from various ailments such as anemia, cancer, GI problems by consuming BG. The aim of our research was to validate the claims of its medicinal values such as chemo-protective action, high anti-oxidants, RBC membrane stabilization activity, and toxicity level.

**Methods:**

Extracts of hexane, ethyl acetate and methanol were quantitatively estimated for total phenolic contents (TPC) and total flavonoid contents (TFC). The same extracts were assessed for their antioxidative potentials with the use of DPPH free radical scavenging assay followed by determination of HRBC membrane stabilization method, Brine Shrimp Lethality Assay (BSLA) and GC-MS analysis.

**Results:**

All the extracts showed high TPC and TFC along with the stronger correlation with the antioxidant activity of the extracts suggesting phenolics and flavonoids contents of the extract might be attributed to showing antioxidant activity. The methanolic and ethyl acetate extracts of the plant also showed remarkable anti-inflammatory activity where methanolic extracts had the lowest EC50. During Brine Shrimp Lethality Assay, all extracts of BG were found to be bioactive and the degree of lethality was found to be concentration dependent. The GC-MS analysis of the methanolic extract of BG revealed 23 compounds which are reported to possess different biological activities.

**Conclusion:**

The study reveals the strong antioxidant and RBC membrane stabilization activity of BG. The Brine Shrimp Lethality Assay found extracts to be bioactive suggesting extracts as a promising candidate for plant-derived anti-tumor compounds. Further, studies are needed to validate the data on cancer cell lines.

## Background

Oxidative stress is the disturbance in the balance between the production of reactive oxygen species, ROS (free radicals) and antioxidant defenses [1]. ROS might be involved as initiators and mediators in several disease such as heart diseases, endothelial dysfunction, atherosclerosis and other cardiovascular disorders, inflammation, brain degenerative impairments, diabetes and eye disease [2]. Humans are in continuous exposure to free radicals produced from exposure of cigarette smoking, alcohol, radiation, or environmental toxins. A biological antioxidant has been defined as any substance that is present at low concentrations compared to an oxidizable substrate and significantly delays or prevents the oxidation of that substrate [3]. Various anti-oxidants have found to possess properties such as anti-atherosclerotic, antitumor, anti-mutagenic, anti-carcinogenic to name a few selected ones [4]. However, studies have reported that some of most commonly used synthetic antioxidants such as Tert-butyl hydroxy anisole (BHA), tert-butyl hydroxytoluene (BHT) are tumor promoters and can induce impairment in blood clotting [5], therefore research has been directed towards plant derived natural antioxidants.

Inflammation is a complex process, which is frequently associated with pain and involves occurrences such as: the increase of vascular permeability, increase of protein denaturation and membrane alteration. NSAIDS are widely used for their anti-inflammatory, analgesic and antipyretic activity and are among the most widely used drugs worldwide [6]. However, these are associated with an increased risk of adverse gastrointestinal, renal and cardiovascular effects [6]. Various natural compounds with promising in vitro and in vivo anti-inflammatory activities have been reported in literature which can be used as novel therapeutic approach for treatment of inflammatory conditions [7].

Brine Shrimp Lethality Bioassay (BSLB) can provide an indication of possible cytotoxic principles in plant extract [8]. This assay has been extensively used for different studies such as for preliminary toxicity screening of plant extracts, detection of fungal toxins, plant extract toxicity, heavy metals, cyanobacteria toxins, pesticides, and cytotoxicity testing of dental materials [9]. Studies have found very good relationship between this simple, inexpensive, and bench-top assay and the antitumor potential of the cytotoxic compounds [10]. So, BSLB might be helpful as a preliminary screening in the antitumor drug designing and synthesis expeditions [10].

Barley Grass (BG) is the leaf portion of the *Hordeum vulgare L.,* also known as barley*,* a member of Poeacea family. Young BG has found to have different nutritional content than of the mature barley grain [11]. The variation in nutritional content of BG may depend on the origin of the plants, soil quality and harvest technique [12]. Barley Grass are rich in dietary minerals such as sodium, magnesium, iron, copper and phosphorus and vitamins such as thiamine, riboflavin, tocopherols and tocotrienols, biotin, folic acid and pantothenic acid [13]. These are found to be richer than those found in some popular vegetables (spinach, tomato, lettuce), fruits (banana) and cow’s milk [13].

In Nepal, the pressed juice of BG is very popular among residents as ‘Jamara Ko Juice’. Various testimonies of people being healed from various ailments such as anemia, cancer, GI problems by consuming BG can be found in the public. For drinking pressed juice, harvesting is usually performed at 7th day. Barley Grass harvesting can be done when the leaves are 12 to 14 in. long to derive the maximum benefits from the grass [11]. Barley Grass are widely accepted as a source of anti-oxidants and various compounds with anti-oxidant activity have been isolated from young barley [14]. Various human and animal studies have reported its beneficial effects such as antiulcer, antioxidant, hypolipidemic, antidepressant, antidiabetic effects and laxative effect [15–19]. Based on the traditional ethnomedicines and existing literatures, BG maximizes the chance of providing novel compounds with promising cytotoxic and anti-oxidant activities. The present study was aimed to evaluate the antioxidant activity, RBC membrane stabilization activity, lethality assay and to evaluate the total phenolic contents of BG.

## Methods

### Chemicals

Gallic acid (GA), ascorbic acid (AA), DPPH and quercetin were purchased from Hi-Media Lab (Mumbai, India). FC reagent and aluminum chloride (AlCl_3_) were purchased from Thermo Fisher Scientific India Pvt. Ltd. (Mumbai, India). Reference standard Diclofenac was obtained from Lomus Pharmaceuticals Pvt. Ltd. (Kathmandu, Nepal). All other chemicals were of standard analytical grade.

### Plant materials

The barley seeds were procured from the local market and were sown in soil from local nursery with daily watering. The Barley Grass were harvested on 7th day of sowing at the month of July. The samples were authenticated by Ganga Datt Bhatt, Research Officer, National Herbarium and Plant Laboratories (NHPL) (Godawari, Lalitpur, Nepal) Voucher number:217. The voucher specimen of this material has been deposited in National Herbarium and Plant Laboratories (NHPL) (Godawari, Lalitpur, Nepal).

### Preparation of the extracts

The harvested BG were washed well using distilled water and shade dried for 21 days before grinding to fine powder. Three hundred grams of fine powder was subjected to successive maceration starting from hexane to ethyl acetate to methanol, 500 ml each for 48 h at room temperature (27 ± 1 °C). The extracts were filtered using a Buckner funnel and Whatman No. 1 filter paper. These extracts were dried in a rotary evaporator under reduced pressure until dryness and stored at 4 °C, protected from light and humidity for further analysis.

### Determination of Total phenolic content

The total phenolic content (TPC) of the extracts was estimated by Folin-Ciocalteu reagent (FCR) method [20] with slight modifications. Briefly, 1 ml of various extracts (1 mg/ml) was mixed with FCR (5 ml, 1:10 v/v DW) and aq. sodium carbonate (4 ml, 7%) solution. The mixture was then incubated for 30 min at 40 °C in a water bath before measuring the absorbance at 760 nm using Microprocessor UV-Vis spectrophotometer-2371 (Electronics India, Himachal Pradesh, India). The phenolic contents were calculated using a standard curve for gallic acid (GA) (10-200 μg/ml), and the result was expressed as mg GAE per gram dry weight of fraction (mg GAE/g). All measurements were performed in triplicates.

### Determination of Total flavonoid content

The total flavonoid content (TFC) was determined by AlCl_3_ coulometric method [21]. An aliquot of 1 ml of various extracts in methanol was added to 10 ml volumetric flask containing 4 ml of distilled water. At the zero-time, 0.3 ml, 5% sodium nitrite was added to the flask. After 5 min, 3 ml of 10% AlCl_3_ was added to the flask. At 6 min, 2 ml of 1 M sodium hydroxide was added to the mixture. Immediately, the total volume of the mixture was made up to 10 ml by the addition of 2.4 ml distilled water and mixed thoroughly. Absorbance of the pink colored mixture was determined at 510 nm against a blank containing using Microprocessor UV-Vis spectrophotometer-2371 (Electronics India, Himachal Pradesh, India). The flavonoid contents were calculated using a calibration curve prepared for Quercetin standards (10 to 100 μg/ml) and the result was expressed as mg of quercetin equivalent/g of extract (mg QE/g of extract).

### Determination of anti-oxidant activity

The DPPH scavenging activity of different fractions was evaluated according to the method of Brand-Williams et al. [22] 1 mL of 0.1 mM DPPH solution in methanol was mixed with 1 mL of each extracts at varying concentrations (5, 10, 15, 20, 25 μg/ml). The corresponding blank sample was prepared, and ascorbic acid (AA) was used as reference standard. Mixture of 1 mL extract and 1 mL DPPH solution was used as control. The mixture was shaken well and incubated for 30 min in the dark. The reaction was carried out in triplicate, and the decrease in absorbance was measured at 517 nm after incubation using a using Microprocessor UV-Vis spectrophotometer-2371 (Electronics India, Himachal Pradesh, India). The scavenging activity was expressed as IC50 (μg/mL). The % scavenging was calculated using the formula:

$$ \%\mathrm{Scavenging}=\left[\left({\mathrm{A}}_0-\mathrm{A}1\right)/{\mathrm{A}}_0\right]\times \kern0.37em 100 $$

Where, A_0_ = absorbance of the control solution.

A_1_ = absorbance of extract/standard.

### Determination of RBC membrane stabilization activity

RBC membrane stabilization activity of three different extracts of BG was evaluated by using in vitro human red blood cell stability method. The membrane stabilizing activity of the sample was assessed according to the method described by Shinde et al [23] with slight modifications.

The assay mixture contained 1 ml phosphate buffer [PH 7.4, 0.15 M], 2 ml hypo saline [0.36%], 0.5 ml HRBC suspension [10% v/v] with 0.5 ml of plant extracts and standard drug diclofenac sodium of various concentrations (10, 20, 40, 80, 100 μg/ml). The control sample consisted of 0.5 mL of RBCs mixed with hypotonic-buffered saline alone. The mixture was incubated at 37 °C for 30 min and centrifuged at 3000 RCF. The hemoglobin content in the suspension was estimated using Microprocessor UV-Vis spectrophotometer-2371 (Electronics India, Himachal Pradesh, India).

$$ \%\mathrm{Protection}=1-\left[\mathrm{OD}\ \mathrm{of}\ \mathrm{Test}/\mathrm{OD}\ \mathrm{of}\ \mathrm{Control}\right]\ \mathrm{X}\ 100 $$

### Determination of toxicity

The toxic activity of the plant was evaluated using Brine shrimp lethality bioassay (BSLA) method [8] where 6 graded doses (viz 1600 μg/mL, 800 μg/mL, 400 μg/mL, 200 μg/mL, 100 μg/mL, and 50 μg/mL) were used. Brine shrimps (*Artemia salina* Leach) nauplii were used as test organisms. For hatching, eggs were kept in artificial sea salt with a constant oxygen supply for 48 h. The mature nauplii were then used in the experiment. DMSO was used as a solvent and also as a negative control. Vincristine sulfate was used as a reference standard in this case. The numbers of survivors were counted after 24 h. Larvae were considered dead if they did not exhibit any internal or external movement during several seconds of observation. The larvae did not receive food. To ensure that the mortality observed in the bioassay could be attributed to bioactive compounds and not to starvation; we compared the dead larvae in each treatment to the dead larvae in the control.

The median lethal concentration (LC50) of the test samples were calculated using the Probit analysis method described by Finney [24], as the measure of toxicity of the plant extract.

$$ \mathrm{Mortality}\%=\left(\mathrm{No}.\mathrm{of}\ \mathrm{dead}\ \mathrm{larvae}/\mathrm{Total}\ \mathrm{no}.\mathrm{of}\ \mathrm{larvae}\right)\times 100. $$

### Gas chromatography-mass spectroscopy analysis

GC-MS analysis was performed at Nepal Academy of Science & Technology (Khumaltar, Kathmandu, Nepal). For GC-MS analysis of plant extract, GC-MS QP2010 (Shimadzu, Kyoto, Japan) equipped with RTx-5MS fused silica capillary column of 30 m length X 0.25 mm diameter X 0.25 μm film thickness. Helium (> 99.99% purity) with 36.2 cm/sec linear velocity was employed as carrier gas. The system was programmed with 3.9 ml/min of total flow rate, 0.95 ml/min of column flow and 3.0 ml/min purge flow. The volume of injected sample was 1 μl. The injector was set in spitless mode having 280 °C of temperature. Oven temperature started from 100 °C and increased to 250 °C at 15 °C/min with holding time of 1 min, which afterwards increased to 280 °C at 30 °C/min with holding time of 1 min and again increased from 280 °C to 300 °C at 15 °C/min with holding time of 11 min.

The ion source temperature and interface temperature were set to 200 °C and 280 °C respectively with solvent cut time of 3.5 min. Total run time was 20 min with mass range scan of 40 to 500 m/z. Identification of compounds was performed by comparing their mass spectra with data from NIST08 mass spectral library.

### Statistical analysis

Each sample analysis was performed in triplicate. All results presented are means (±SEM) of at least three independent experiments. Statistical analysis, ANOVA with a statistical significance level set at *p* < 0.05 with post-hoc Tukey procedure was carried out with SPSS 16 for Windows. Correlations between the total phenolic contents, flavonoid contents and antioxidant capacities were determined using the Pearson correlation.

## Results

### Total phenolic content determination

**Fig. 1:**
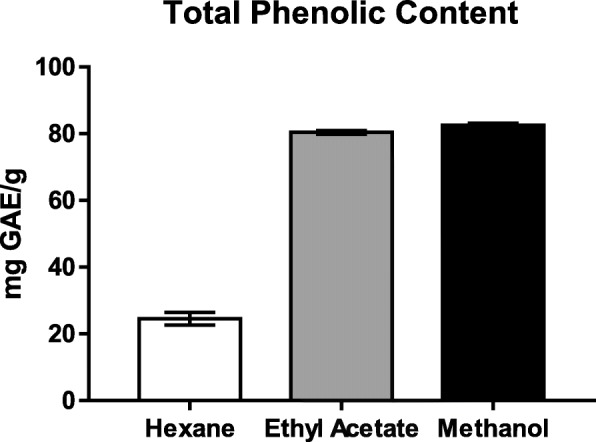
TPC of various extracts of BG

The total phenolic content of three extracts determined by FCR method were expressed as GAE/g dried extract (Fig. 1). The phenolic content in all extracts ranged from 24.55 to 82.56 mg GAE/g dried extracts representing an approximate three-fold variation (Table 1). Methanolic extract had significantly higher phenolic contents than ethyl acetate and hexane.

**Table 1:** TFC and TPC of various extracts of BG

Extracts	TFC (mg QE/ g dried extract)	TPC (mg GAE/ g dried extract)
Hexane	18.94 ± 0.42^a^	24.55 ± 1.94^a^
Ethyl Acetate	19.07 ± 0.33^b^	80.42 ± 0.54^b^
Methanol	45.76 ± 2.40^b^	82.56 ± 0.59^b^

Values are in Mean ± SEM (n = 3). Values in the same column followed by a different letter are significantly different (*P* < 0.001)

### Total flavonoid content determination

**Fig. 2:**
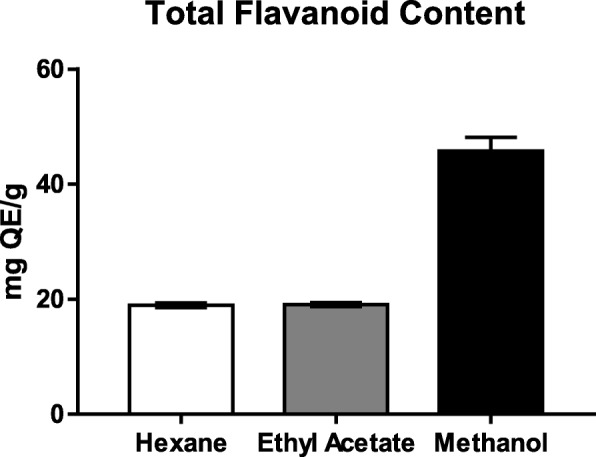
TFC of various extracts of BG

The result of total flavonoid contents of three extracts of barley grass is given in Fig. 2. The total flavonoid contents were reported as QE, ranged from 18.94 to 45.76 mg QE/g dried extracts (Table 1). Methanolic extract had significantly highest flavonoid content followed by ethyl acetate and hexane extracts.

### Anti-oxidant activity determination

**Fig. 3:**
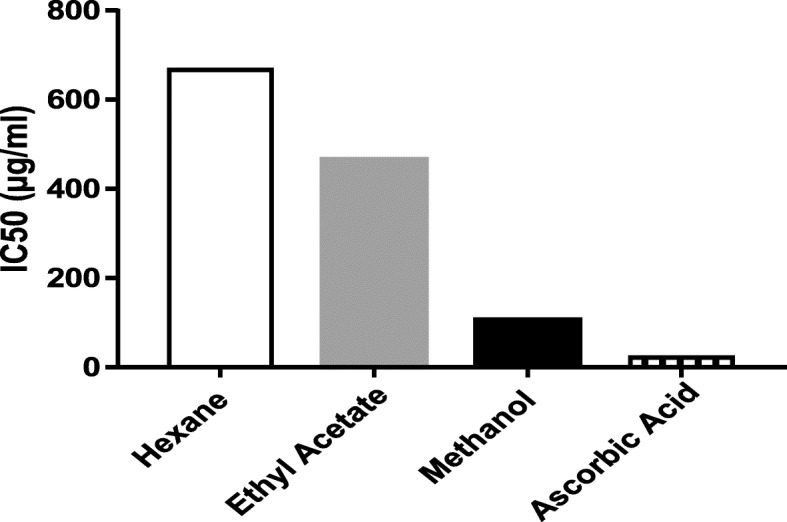
IC_50_ values of various extracts and ascorbic acid

The anti-oxidant potential of all extracts were assessed by DPPH free radical scavenging assay. The radical scavenging is one of mechanism of anti-oxidant activity. The results were expressed in terms of IC_50_ and is shown in Table 2. The lower IC_50_ represents higher scavenging ability. The IC_50_ of methanolic extract (IC_50_ = 104.9 μg/ml) was found to be significantly lower than the ethyl acetate (455.24 μg/ml) and hexane (659.97 μg/ml) extracts. However, the activity of all extracts was found to be less when compared to standard, AA (22.58 μg/ml) (Fig. 3).

**Table 2:** IC_50_ Values of different extracts and ascorbic acid

Extracts	Regression Equation	Correlation Coefficient (R^2^)	IC_50_ (μg/ml)
Hexane	y = 0.0758x - 0.0257	0.9651	659.97
Ethyl Acetate	y = 0.1111x - 0.5772	0.9347	455.24
Methanol	y = 0.5009x- 2.5436	0.9777	104.90
AA	y = 2.7378x - 11.819	0.9838	22.58

*AA* Ascorbic acid

### Correlation between TPC, TFC and anti-oxidant activity of the extracts

There was a higher correlation between total flavonoid content (TFC) and DPPH radical scavenging activity (R = − 0.936). Similarly, the correlation between total phenolic content (TPC) and DPPH radical scavenging activity (R = − 0.795) was also higher.

### RBC membrane stabilization activity determination

**Fig. 4:**
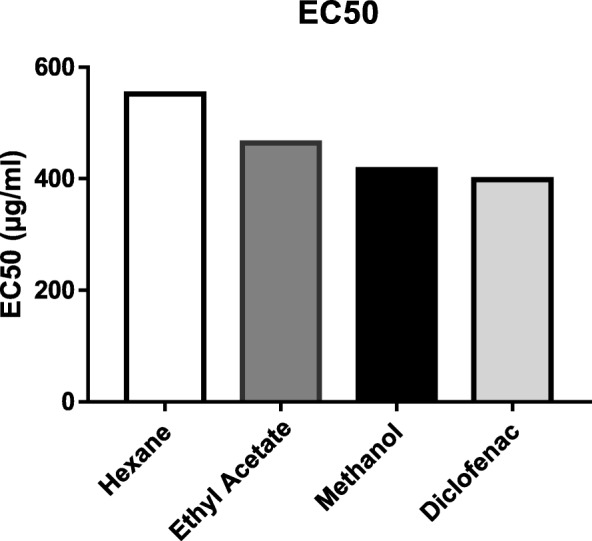
EC_50_ values of various extracts and standard (Diclofenac)

Membrane stabilizing activity was assayed to evaluate the inhibition of hypotonic solution induced lysis of human erythrocyte membrane. The extracts were effective in inhibiting the hypotonicity induced hemolysis at different concentrations. These provides evidence for membrane stabilization as a possible mechanism of their anti-inflammatory effect. The EC50 found to be in order of Hexane> Ethyl acetate> Methanol> Diclofenac (Fig. 4; Table 3). Significant differences (*p* < 0.005) was found between % protection values of different extracts.

**Table 3:** EC_50_ values of different extracts and diclofenac

Extracts	EC_50_ (μg/ml)
Hexane	551.69
Ethyl Acetate	464.40
Methanol	416.15
Diclofenac	398.31

### Determination of toxicity

All the extracts were subjected to Brine Shrimp lethality bioassay for possible toxic action. In this study, methanol extract was found to be the most toxic to Brine Shrimp nauplii, with LC50 of 266.49 μg/ml whereas anticancer drug, vincristine sulphate showed LC50 value 1.707 μg/ml (Table 4). The order at which cytotoxic potential of the test samples was as follows: Vincristine sulphate> Methanol> Hexane> Ethyl acetate.

**Table 4:** LC_50_ of the different extracts Brine shrimp lethality bioassay

Test Sample	Concentration (μg/ml)	%Mortality	LC_50_ (μg/ml)
Hexane	50	20	290.72
	100	30	
	200	50	
	400	60	
	800	70	
	1600	90	
Ethyl Acetate	50	10	367.91
	100	20	
	200	50	
	400	50	
	800	70	
	1600	80	
Methanol	50	0	266.49
	100	30	
	200	50	
	400	60	
	800	80	
	1600	90	
VS	0.25	30	1.71
	0.5	40	
	1	50	
	5	90	
	10	100	

VS *Vincristine Sulphate*

### Gas chromatography-mass spectroscopy analysis

**Fig. 5:**
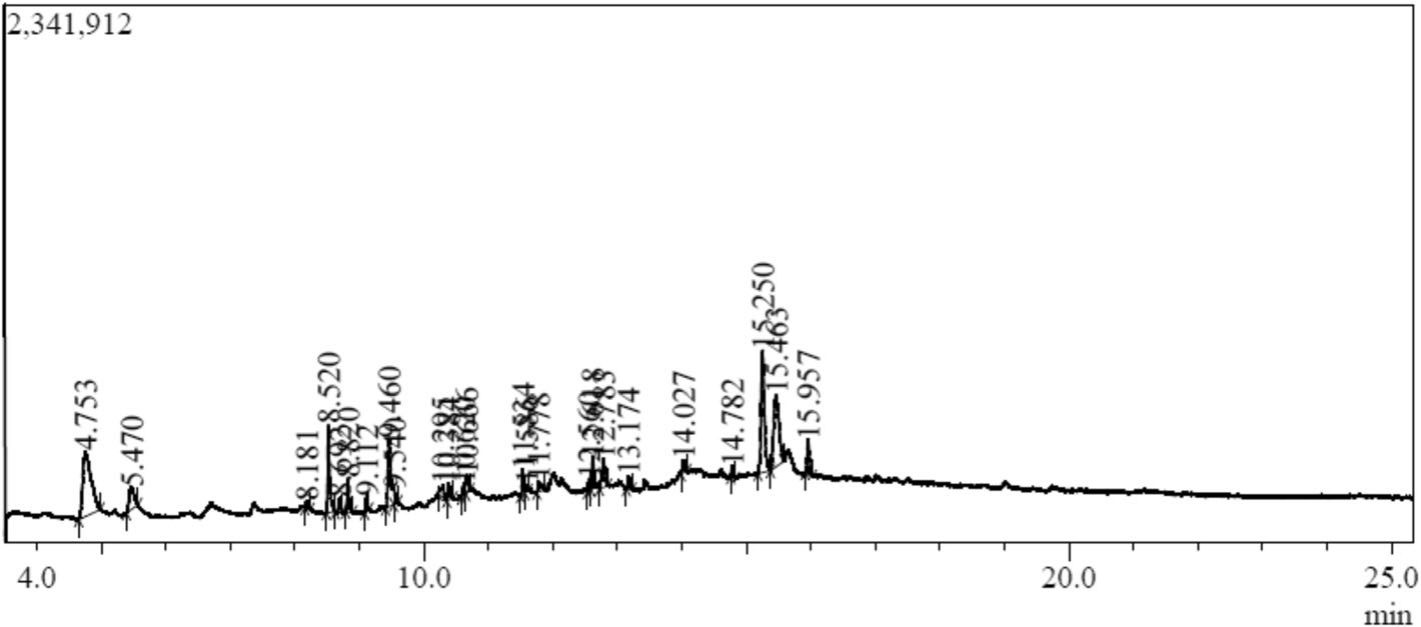
GCMS chromatogram of methanolic extracts of BG

The GC-MS analysis of phytoconstituents in methanolic extract of barley grass revealed the presence of twenty-three major phytoconstituents (Fig. 5; Table 5). The major phytocomponents reported are Indolizine (21.78%), Octadecyl trifluoroacetate (15.85%), Palmitic acid (8.15%),1-Hexadecyne (6.98%), 1H-Indole,5-methyl- (4.46%), 9,12,15-Octadecatrienoic acid (1.64%), Phytol (1.61%) and Squalene (0.82%) (Figure S1).

**Table 5:** Composition of methanolic extract of BG

Peak#	R. T	Area%	Name	Mol. Wt.
1	4.753	21.78	Indolizine	117
2	5.470	4.46	1H-Indole, 5-methyl-	131
3	8.181	0.53	Hexadecen-1-ol, trans-9-	240
4	8.520	10.35	1-Hexadecyne	222
5	9.112	0.94	Hexadecanoic acid, methyl ester	270
6	9.460	8.15	n-Hexadecanoic acid	256
7	9.540	0.93	1-Tridecyn-4-ol	196
8	10.295	1.65	11,14,17-Eicosatrienoic acid, methyl ester	320
9	10.384	1.61	Phytol	296
10	10.620	1.26	5-Tetradecyne	194
11	10.666	1.64	9,12,15-Octadecatrienoic acid, (Z,Z,Z)-	278
12	11.534	1.59	Octanoic acid, 2-dimethylaminoethyl ester	215
13	11.586	0.40	Oxalic acid, propyl tridecyl ester	314
14	11.778	1.24	(E)-13-Docosenoic acid	338
15	12.560	0.77	3-Cyclopentylpropionic acid, 2-dimethylami	213
16	12.618	2.11	3-Cyclopentylpropionic acid, 2-dimethylami	213
17	12.783	3.20	Hexadecen-1-ol, trans-9-	240
18	13.174	0.99	Diisooctyl phthalate	390
19	14.027	1.12	Heptadecyl heptafluorobutyrate	452
20	14.782	0.82	Squalene	410
21	15.250	15.85	Octadecyl trifluoroacetate	366
22	15.463	15.71	4-Oxo-2-phenyl-1,4-dihydroquinoline-3-carbonitrile	246
23	15.957	2.92	1-Heptadecanol, acetate	298

R.T *Retention Time (minutes)*

## Discussion

Phenolic compounds are a group of chemical compounds that are widely distributed in nature. Phenolic compounds are nutritionally important and the interest in these compounds are increasing for their various bioactivities such as antioxidant, anti-aging, anti-inflammatory and anti-proliferative activities [25]. We found methanol to be significantly more efficient to extract polyphenolic compounds compared to ethyl acetate and hexane extracts of BG. These findings are in support of higher solubility of phenols in polar solvents providing high concentration of these compounds in the extracts obtained using polar solvents for the extraction [26]. Different phenolic compounds including flavones (e.g. major leaf antioxidants, such as saponarin, lutonarin, and 2-O-glucosylvitexin), leucoanthocyanidins, catechins, and coumarins have been found in young barley extracts [27]. The TPC contents in BG juice were significantly higher than wheatgrass and rice juices reported by Wangcharoen et al. [28]. However, phenolic contents in BG can be affected by different factors such as light quality, cultivars and harvesting times [29, 30].

Flavonoids are some of the most common phenolics, widely distributed in the plant tissues. Reviews on flavonoids have found it as a possible cancer-preventive agent [31]. Quercetin, a flavonoid, can be considered as the prototype of a naturally occurring chemo-preventive agent [32]. In this study, the total flavonoids contents of the different organic crude plant extracts were determined as quercetin equivalents by a modified aluminum chloride coulometric method [21]. Methanolic extract found to have significantly higher flavonoid content than ethyl acetate and hexane extract.

The antioxidant activity was evaluated by the capacity of antioxidant compound to reduce the DPPH radical as indicated by the decrease in its absorbance at 517 nm until the reaction reached a plateau. Significant differences (*p* < 0.0383) was obtained between antioxidant activity of the different extracts of BG. The methanolic extracts of BG had lowest IC50 value and thus with the highest antioxidant activity followed by ethyl acetate and hexane. The IC50 value of methanolic extract was found to be 104.41 μg/ml which is similar to the IC50 found by the Nepal et al. for 80% methanolic extract [33]. The differences of antioxidant activity between various extracts could be due to the difference in total amount of phenolics and flavonoids as phenolic and flavonoids are reported to have anti-oxidant activity [34] [35]. Pearson Correlation analysis were used to determine the relation between these parameters. There was a higher correlation between TFC and DPPH radical scavenging activity (R = − 0.936), and the correlation between TPC and DPPH radical scavenging activity (R = − 0.795) was also found to be high suggesting phenolics and flavonoids might have attributed to show anti-oxidant activity in BG. The correlation was found to be negative as increase in TPC and TFC caused increase in antioxidant activities, which was exposed by lower IC50 of DPPH scavenging activity. Previous studies have also showed that total phenolic contents of culinary plants were significantly correlated (*p* < 0.05) to their antioxidant activities [36].

In RBC membrane stabilization activity test, all extracts were effective in inhibiting the hypotonicity induced hemolysis at different concentrations. The methanolic extract had lowest EC50 than ethyl acetate and hexane. RBC membrane stabilization activity test can be related to the anti-inflammatory activity of the BG. This is by far the first reported study on HRBC membrane stabilization study on BG. The GC-MS analysis of methanolic extract reported several phytoconstituents with anti-inflammatory activity such as Indolizine [37], 9,12,15-Octadecatrienoic acid [38], Phytol [39], Squalene [40]. The presence of such compounds could be the reason for the activity of extracts.

The GC-MS analysis of methanolic extract of BG revealed 23 compounds. These compounds are reported to possess different activities. For ex, Indolizine has anti-inflammatory properties [37]. Phytol is a diterpene which is reported to have anti-inflammatory and cancer preventive properties [39]. Fatty acids like 13-docosenic acid and 9,12,15-Octadecatrienoic acid are reported to be in BG. They have cancer preventive, nematicide, anti-arthritic, anti andrigenic, anti-infammatory and hypocholesterolemic properties [38]. Cyclotetracosane has anti-diabetic or alpha amylase activity [41]. Squalene possess anti-bacterial, anti-oxidant, cancer preventive, anti-tumor and lipoxygenase inhibitor [40]. Hexadecen-1-ol, trans 9 possess anti-oxidant and anti-tumor [42].

The degree of lethality shown by BG was found to be directly proportional to the concentration of the extractives ranging from the lowest concentration (50 μg/ml) to the highest concentration (1600 μg/ml). This concentration dependent increment in percent mortality of Brine Shrimp nauplii produced by the BG may indicates the presence of cytotoxic principles in these extracts.

Methanol extracts had the lowest LD50 as 266.49 μg/ml followed by ethyl acetate, 367.91 μg/ml and hexane, 290.72 μg/ml. In toxicity evaluation of plant extracts by Brine shrimp lethality bioassay LD50 values lower than 1000 μg/ml are considered bioactive [8]. Thus, all extracts of BG are found to be bioactive. The brine shrimp assay is significantly correlated with in vitro growth inhibition of human solid tumor cell lines demonstrated by the national Cancer Institute (NCI, USA) and it can show the value of this bioassay as a pre-screening tool for antitumor drug research [43]. Therefore, these extracts can be regarded as promising candidate for plant derived anti-tumor compounds. A study on a barley grass supplement named as Herb-All Barley Powder found the LD50 to be 448.42 ppm in a similar setting [44].

## Conclusions

This study showed the importance of BG and its possible health benefits. Barley Grass could be considered as functional drinks with antioxidant potential because of their higher phenolic content and flavonoid content. There was a strong correlation of TFC, TPC and anti-oxidant activity of the extracts which suggests the flavonoids and phenolics might have shown anti-oxidant activity in these extracts. Presence of anti-inflammatory compounds and because of significant RBC membrane stabilization activity, BG can also be regarded as functional drinks with anti-inflammatory potential. All extracts of BG had shown significant bioactivity towards brine shrimp which have good correlation with tumor cell lines suggesting these extracts to be as promising candidate for plant derived anti-tumor compounds. Thus, further studies are needed to validate the data on cancer cell lines.

## Supplementary information

**Additional file 1 : Figure S1.** Chromatogram of various compounds in methanolic extract of BG based on GC-MS profile of Fig. 6. **Table S1.** Absorbance values of extracts in TPC determination. **Table S2.** Absorbance values of extracts in TFC determination. **Table S3.** Absorbance values of extracts and ascorbic acid in anti-oxidant activity determination. **Table S4.** Absorbance values of extracts and diclofenac in anti-Inflammatory activity determination. **Table S5.** Dead brine shrimp counts of extracts in Brine shrimp lethality assay.

## Data Availability

The datasets used and/or analyzed during the current study are available from the corresponding author on reasonable request.

## Abbreviations

AlCl_3_: Aluminum chloride
BG: Barley grass
BSLA: Brine shrimp lethality assay
DMSO: Dimethyl sulfoxide
DPPH 1: 1 -diphenyl-2-picryhydrazyl
FCR: Folin- ciocalteu reagent
GAE: Gallic acid equivalent
GAE: Gallic Acid equivalents
GC-MS: Gas chromatography mass spectroscopy
HRBC: Human red blood cell
QE: Quercetin equivalent
TFC: Total flavonoids content
TPC: Total phenolics content
