# Supplementary material for: Bioactivity evaluations of leaf extract fractions from young barley grass and correlation with their phytochemical profiles
Source: BMC Complement Med Ther. 2020 Feb 28;20:64. doi: 10.1186/s12906-020-2862-4 (PMC7076879; doi:10.1186/s12906-020-2862-4)
Supplement: Supplementary file 1 — Additional file 1 : Figure S1. Chromatogram of various compounds in methanolic extract of BG based on GC-MS profile of Fig. 6. Table S1. Absorbance values of extracts in TPC determination. Table S2. Absorbance values of extracts in TFC determination. Table S3. Absorbance values of extracts and ascorbic acid in anti-oxidant activity determination. Table S4. Absorbance values of extracts and diclofenac in anti-Inflammatory activity determination. Table S5. Dead brine shrimp counts of extracts in Brine shrimp lethality assay. [file 12906_2020_2862_MOESM1_ESM.docx]

**Supplementary Materials**

**Bioactivity Evaluations of Leaf Extract Fractions from Young Barley Grass and Correlation with Their Phytochemical Profiles**

Mamata Panthi^1⸸^, Romit Kumar Subba^1,2⸸^, Bechan Raut^1^, Dharma Prasad Khanal^1^ and Niranjan Koirala^2*^

^1^Department of Pharmacy, Manmohan Institute of Health Sciences, Tribhuvan University, Kathmandu, Nepal; [mamta.panthi1234@gmail.com](mailto:mamta.panthi1234@gmail.com) (M.P), [rautbechan@yahoo.com](mailto:rautbechan@yahoo.com) (B.R); [drdharmakhanal@gmail.com](mailto:drdharmakhanal@gmail.com) (DPK)

^2^ Department of Natural Products Research, Dr. Koirala Research Institute for Biotechnology and Biodiversity, Kathmandu, Nepal

Address for correspondence: [koirala.biochem@gmail.com](mailto:koirala.biochem@gmail.com) (N.K)

^⸸^ These authors equally contributed to this manuscript.

**Figure S1.** Chromatogram of various compounds in methanolic extract of BG based on GC-MS profile of Figure 6.

1. Indolizine

1. 1H-Indole, 5-methyl

1. Hexadecen-1-ol, trans-9-

1. 1-Hexadecyne

1. Palmitic acid

1. 1-Tridecyn-4-ol

1. 11,14,17-Eicosatrienoic acid, methyl ester

1. Phytol

1. 5-Tetradecyne

1. Linolenic acid

1. Octanoic acid, 2-dimethylaminoethyl ester

1. Oxalic acid, propyl tridecyl ester

1. (E)-13-Docosenoic acid

1. 3-Cyclopentylpropionic acid, 2-dimethylaminoethyl ether

1. Hexadecen-1-ol, trans-9

1. Diisooctyl phthalate

1. Heptadecyl heptafluorobutyrate

1. Squalene

1. Octadecyl trifluoroacetate

1. 4-Oxo-2-phenyl-1,4-dihydroquinoline-3-carbonitrile

1. 1-Heptadecanol, acetate

**Data Values of Various Tests**

**Table S1.** Absorbance values of extracts in TPC determination

| Extracts | Absorbance |
| --- | --- |
| Hexane | 0.219, 0.284, 0.251 |
| Ethyl Acetate | 0.794, 0.802, 0.784 |
| Methanol | 0.814, 0.804, 0.824 |

**Table S2.** Absorbance values of extracts in TFC determination

| Extracts | Absorbance |
| --- | --- |
| Hexane | 0.083, 0.084, 0.089 |
| Ethyl Acetate | 0.085, 0.084, 0.089 |
| Methanol | 0.224, 0.189, 0.197 |

**Table S3.** Absorbance values of extracts and ascorbic acid in anti-oxidant activity determination.

absorbance of control: 0.783

| Conc. (µg/ml) | Hexane | Ethyl Acetate | Methanol | Ascorbic Acid |
| --- | --- | --- | --- | --- |
| 5 | 0.780, 0.7802, 0.781 | 0.783, 0.782, 0.7821 | 0.779, 0.7793, 0.780 | 0.763, 0.738, 0.755 |
| 10 | 0.777, 0.7771, 0.776 | 0.780, 0778, 0.779 | 0.771, 0.771, 0.770 | 0.671, 0.66, 0.656 |
| 15 | 0.775, 0.7743, 0.774 | 0.776, 0.775, 0.775 | 0.763, 0.735, 0.737 | 0.585, 0.570, 0.559 |
| 20 | 0.770, 0.771, 0.769 | 0.771, 0.777, 0.770 | 0.725, 0.713, 0.721 | 0.480, 0.450, 0.447 |
| 25 | 0.770, 0.769, 0.7691 | 0.763, 0.764, 0.7635 | 0.705, 0.708, 0.707 | 0.317, 0.310, 0.306 |

**Table S4.** Absorbance values of extracts and diclofenac in anti-Inflammatory activity determination.

Absorbance of Control: 0.732

| Conc. (µg/ml) | Hexane | Ethyl Acetate | Methanol | Diclofenac |
| --- | --- | --- | --- | --- |
| 10 | 0.67, 0.673, 0.676 | 0.673, 0.671, 0.675 | 0.622, 0.625, 0.618 | 0.596, 0.595, 0.593 |
| 20 | 0.659, 0.655, 0.662 | 0.654, 0.659, 0.661 | 0.615, 0.614, 0.611 | 0.591, 0.59, 0.592 |
| 40 | 0.649, 0.655, 0.651 | 0.637, 0.633, 0.637 | 0.61, 0.607, 0.606 | 0.576, 0.578, 0.579 |
| 80 | 0.637, 0.634, 0.638 | 0.622, 0.626, 0.619 | 0.578, 0.577, 0.58 | 0.562, 0.559, 0.557 |
| 100 | 0.611, 0.619, 0.615 | 0.607, 0.611, 0.605 | 0.564, 0.567, 0.561 | 0.539, 0.54, 0.538 |

**Table S5.** Dead brine shrimp counts of extracts in Brine shrimp lethality assay.

| Conc. (µg/ml) | Hexane | Ethyl Acetate | Methanol |
| --- | --- | --- | --- |
| 50 | 2 | 1 | 0 |
| 100 | 3 | 2 | 3 |
| 200 | 5 | 5 | 5 |
| 400 | 6 | 5 | 6 |
| 800 | 7 | 7 | 8 |
| 1600 | 9 | 8 | 9 |
